# Nitrogen gas flushing can be bactericidal: the temperature-dependent destiny of *Bacillus weihenstephanensis* KBAB4 under a pure N_2_ atmosphere

**DOI:** 10.3389/fmicb.2014.00619

**Published:** 2014-11-14

**Authors:** Patricia Munsch-Alatossava, Tapani Alatossava

**Affiliations:** Division of Food Technology, Department of Food and Environmental Sciences, University of HelsinkiHelsinki, Finland

**Keywords:** nitrogen gas, bactericidal, *Bacillus*, *Pseudomonas*, small colony variants

## Abstract

Gram-negative *Pseudomonas* and Gram-positive *Bacillus* are the most common spoilage bacteria in raw and pasteurized milk, respectively. In previous studies, nitrogen (N_2_) gas flushing treatments of raw and pasteurized milk at cold chain-temperatures inhibited bacterial spoilage and highlighted different susceptibilities to the N_2_ treatment with the exclusion of certain bacterial types. Here, we investigated the effects of pure N_2_ gas flushing on representative strains of these genera grown in mono- or co-cultures at 15 and 25°C. *Bacillus weihenstephanensis*, a frequent inhabitant of fluid dairy products, is represented by the genome-sequenced KBAB4 strain. Among *Pseudomonas, P. tolaasii* LMG 2342^T^ and strain C1, a raw milk psychrotroph, were selected. The N_2_ gas flushing treatment revealed: (1) temperature-dependent responses; (2) inhibition of the growth of both pseudomonads; (3) emergence of small colony variants (SCVs) for *B. weihenstephanensis* strain KBAB4 at 15°C induced by the N_2_ treatment or when grown in co-culture with *Pseudomonas* strains; (4) N_2_ gas flushing modulates (suppressed or stimulated) bacterial antagonistic reactions in co-cultures; (5) most importantly, scanning electron microscopy (SEM) and transmission electron microscopy (TEM) analyses revealed that at 25°C the majority of the KBAB4 cells were killed by pure N_2_ gas flushing. This observation constitutes the first evidence that N_2_ gas flushing has bactericidal effects.

## Introduction

The challenges presently faced by food production systems require the urgent development of additional steps to alleviate food-borne pathogens and to tackle food spoilage. In developed and developing countries, as much as 30–40% of the food produced is lost to waste (Godfray et al., 2010). In developing regions, the waste of milk, for example, is relatively high during post-harvest handling and storing (Gustavsson et al., 2011). In developed countries, numerous studies point to the vulnerability of the cold chain for raw or pasteurized milk. Milk is colonized by numerous “psychrotrophic” or “psychrotolerant” bacterial types which can easily grow at refrigeration temperatures and induce spoilage (Cousin, 1982; Hayes and Boor, 2001). Most psychrotrophic bacteria found in raw milk, many of which are Gram negative bacteria, are generally considered as benign; their spoilage features arise from various heat-stable exoenzymes that degrade the different milk components. In addition, the trends in antibiotic resistance levels observed in psychrotrophic bacterial populations over time during the cold storage of raw milk suggest that bacterial growth should be prevented by supplementary means (Munsch-Alatossava and Alatossava, 2007; Munsch-Alatossava et al., 2012a,b). Many studies have examined complementary methods to cold storage to control bacterial growth in highly perishable fluid milk products (Murray et al., 1983; Ruas-Madieodo et al., 1996; Dechemi et al., 2005; Rajagopal et al., 2005).

We investigated at the laboratory scale, the possibility of flushing raw milk with pure N_2_ gas, applied in an “open system.” Treatments at 6, 7, and 12°C hindered bacterial growth and affected the spoilage potential (Munsch-Alatossava et al., 2010a; Munsch-Alatossava and Alatossava, 2011a). More strikingly, some bacterial types were particularly targeted: colonies that correspond to *Bacillus cereus*-type (cultured on Mannitol Egg Yolk Polymyxin B agar plates) proliferated in control conditions during the cold storage of milk; contrarily to flushed raw milk samples, for which *B. cereus*-type colonies, if initially present, were no longer found (Munsch-Alatossava et al., 2010a). Considering only bacteria that are able to grow on egg yolk agar, phospholipases-producers present in raw milk were “sooner or later” excluded from the agar plates under the flushing treatment (Munsch-Alatossava et al., 2010a,b). Among the bacterial types, retrieved from flushed milk on Mac- Conkey agar, lactose non-fermenters seemed to be particularly targeted compared to the controls (Munsch-Alatossava, Gursoy, Alatossava, unpublished data). Noteworthy, the most sensitive groups at the laboratory scale were also hindered by the treatments at the pilot plant scale, when N_2_ gas was extracted and concentrated from the surrounding atmosphere to treat raw milk (Munsch-Alatossava et al., 2011b).

We recently explored the possibility of flushing pasteurized milk following the pasteurization step and observed that bacterial growth was inhibited up to 35 days at 6°C, when the treatment was applied shortly after pasteurization; *Bacillus weihenstephanensis* was frequently isolated from controls or N_2_-flushed pasteurized milk (Munsch-Alatossava et al., 2013). Previous reports indicated that *B. weihenstephanensis* is commonly found in fluid milk (Huck et al., 2009; Ivy et al., 2012). *B. weihenstephanensis* was earlier distinguished from *B. cereus* by its ability to grow at 7 and not at 43°C (Lechner et al., 1998; Von Stetten et al., 1999) observed that *B. weihenstephanensis* was still able to grow at 38°C. The species comprises most of the psychrotolerant strains of *B. cereus*. Based on phenotypic and genotypic features, the bacterium was ascribed to the phylogenetic group VI of *B. cereus* (Guinebretière et al., 2008). Recent reports point to potential health risks. The production of cereulide was observed at 8°C in two *B. weihenstephanensis* environmental strains (Thorsen et al., 2006). The *B. weihenstephanensis* KBAB4 strain, considered to be moderately psychrotolerant, was more virulent at low temperature (15°C) compared to 30°C (Lapidus et al., 2008; Réjasse et al., 2012). A recent report proposed that because, some strains were able to grow at temperatures as low as 6°C and to produce toxin at 8°C, *B. weihenstephanensis* should be considered as potentially hazardous for refrigerated foods (Markland et al., 2013).

To clarify previous observations from the investigations of raw and pasteurized milk microbiota and to increase our knowledge of bacterial inhibition or exclusion under N_2_ treatments, representative Gram-positive and Gram-negative bacteria were studied in single or dual cultures, at two temperatures (15 and 25°C). The combinations were composed of the strain KBAB4 challenged with one of two pseudomonads: *Pseudomonas tolaasii*, a common mushroom pathogen (Rainey et al., 1991) that is occasionally found in milk (Wiedmann et al., 2000), and the *Pseudomonas* spp. raw milk psychrotrophic isolate C1. C1 represents the ideal spoilage candidate because it has protease, lipase, phospholipase activities; it is also haemolytic and has antibiotic multi-resistance features (Munsch-Alatossava and Alatossava, 2006, 2007).

This study was designed to determine whether pure N_2_ flushing has deleterious effects on bacterial strains grown as single or dual cultures, and whether the temperature modulates the effects of the N_2_ flushing. We asked whether sporulation of *B. weihenstephanensis* was promoted by the treatments. We also asked what types of bacterial interactions are present in the co-cultures and whether N_2_ flushing modifies these interactions.

## Materials and methods

### The N_2_ gas flushing system

The gas flushing system has been fully described (Munsch-Alatossava et al., 2010a, 2013). Briefly, the N_2_ gas (99.999% pure N_2_, AGA Ltd, Riihimäki, Finland) flow was adjusted to 120 ml/mn and entered the culture flasks through sterile filters (0.22 μm). Together with the corresponding controls, the flushed flasks were continuously mixed at 220 rpm and were partially immersed in a water bath (MGW Lauda M/S); the temperature inside the flasks was 25 ± 0.1°C or 15 ± 0.1°C (99.5% ethanol was used as a cooling agent).

### Bacterial strains and microbiological studies

*B. weihenstephanensis* strain KBAB4, *P. tolaasii* strain LMG 2342^T^ and the psychrotrophic *Pseudomonas* sp. raw milk isolate C1 were grown on Brain Heart Infusion (BHI, Lab M, Heywood, UK) agar; the strains were appropriately stored and routinely sub-cultured. At the beginning of the experiment, 5 μl of overnight bacterial monocultures (in BHI broth) were added to 100 ml of BHI broth, according to the experimental conditions. The following abbreviations identify the experimental conditions: KC and KN correspond to *B. weihenstephanensis* KBAB4 grown in control and N_2_- flushed cultures, respectively; C1C, 42C, C1N, and 42N correspond to the pseudomonads C1 and LMG 2342^T^ grown in control and flushed cultures, respectively. The dual cultures are described as follows: the viable cells from the control and flushed cultures of KBAB4, grown in co-culture with C1, correspond to K(C1)C and to K(C1)N, respectively; the levels of C1 or 42 in co-culture with KBAB4 correspond to C1(K)C and 42(K)C, C1(K)N and 42(K)N for the controls and flushed co-cultures, respectively.

Following inoculation, at the different sampling times, the N_2_ flow was briefly interrupted and 500 μl of bacterial cultures was retrieved, serially diluted in saline solution (0.85% NaCl), and cultured on BHI agar when KBAB4 was grown alone, or in co-culture with LMG 2342^T^ or C1. The viable cell counts for LMG 2342^T^ and C1 were separately determined on Mac-Conkey agar (Difco, Becton Dickinson, USA). The bacterial counts were determined from triplicate platings after incubation at 30°C for 24 h. Spore levels from cultures, that contained the strain KBAB4 in mono- or co-culture, were determined following a heat treatment of 500 μl of culture samples at 80°C for 12 min and platings on BHI agar. The plates were incubated at 30°C for 24–48 h.

### Electron microscopy of bacterial cells

The analyses were performed at the end of the flushing treatments for both control and flushed cultures of *B. weihenstephanensis* KBAB4 grown as a single culture, at 15 and 25°C. For electron microscopy (EM) analysis, the cells were fixed with 2.5% glutaraldehyde (GA) by adding 110 μl of a 25% GA stock solution [EM-grade, Electron Microscopy Sciences (EMS), Hatfield, PA, USA] to a 1 ml bacterial culture in a 1.5 ml Eppendorf tube. The samples were mixed thoroughly, and kept for 2 h at room temperature (RT) to complete the fixation. The GA-fixed cells were pelleted by centrifugation at 5900× g for 5 min (Eppendorf 5415D), after which the supernatant was removed, and the pellet of the GA-fixed cells was suspended in 1 ml 0.1 M Na-phosphate buffer (pH 6.8) by thorough vortex-mixing. The samples of GA-fixed bacterial cell suspensions were stored at 4°C for scanning electron microscopy (SEM) and transmission electron microscopy (TEM) specimen preparations and analyses at the Electron Microscopy Unit (Institute of Biotechnology, University of Helsinki). Standard specimen preparation techniques for SEM and TEM analysis were used. The SEM specimens of the GA-fixed bacterial cell suspensions were dehydrated with a graded series of ethanol (30, 50, 70, 96, and 100%), dried with hexamethyldisilazane, mounted into aluminum stubs and coated with platinum. The specimens were examined in a Quanta FEG250 (FEI Europe, Eindhoven, Netherlands) scanning electron microscope. The TEM specimens of the GA-fixed bacterial cell suspensions were post-fixed with 1% osmium tetroxide (EMS) for 1 h at RT, followed by dehydration with a graded series of ethanol (30, 50, 70, 96, and 100%) and acetone, and finally embedded gradually in hard Epon resin (TAAB Embedding). Each Epon-embedded specimen was sectioned; sections containing bacterial cell material were further picked up on the Pioloform-coated one hole-type grid and were post-stained with aqueous uranyl acetate and lead citrate. The specimens were examined in a JEM-1400 (Jeol Ltd., Tokyo, Japan) transmission electron microscope.

## Results

### Effect of N_2_ flushing on the growth of bacterial mono- and co-cultures at 25°C

**Figure 1:**
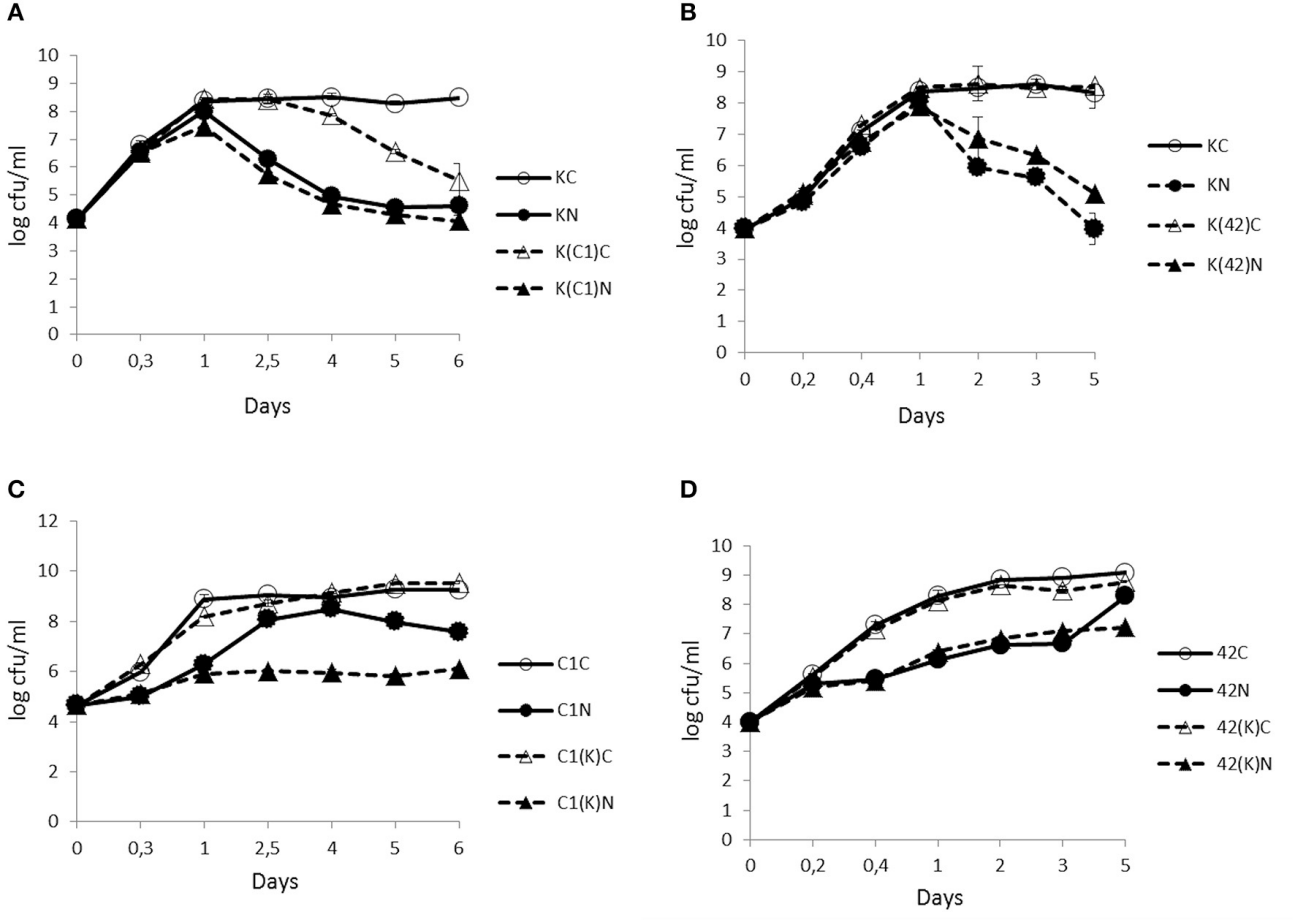
**Time-course analyses of the number (cfu/ml) of *B. weihenstephanensis* KBAB4 cells (K) established as mono- or co-cultures in BHI broth at 25°C with (A) a *Pseudomonas* raw milk isolate C1 (C1) or (B) *P. tolaasii* LMG 2342^T^ (42)**. Similarly, the number (cfu/ml) of **(C)** *Pseudomonas* strain C1 (C1) or **(D)** LMG 2342^T^ (42) cells as mono- or co-cultures with *Bacillus* strain KBAB4 under continuous pure N_2_ gas flushing (N) together with the control conditions **(C)**. Error bars indicate standard deviations. **(A,B)** KC, monoculture of *B. weihenstephanensis* KBAB4 as control; KN, monoculture of *B. weihenstephanensis* KBAB4 flushed with N_2_; K(C1)C, KBAB4 grown in the presence of C1 as control; K(C1)N, KBAB4 grown in the presence of C1 flushed with N_2_; K(42)C, KBAB4 grown in the presence of LMG 2342^T^ as control; K(42)N, KBAB4 grown in the presence of LMG 2342^T^ flushed with N_2_. **(C)** C1C, monoculture of the raw milk isolate C1 as control; C1N, monoculture of the raw milk isolate C1 flushed with N_2_; C1(K)C, C1 grown in the presence of KBAB4 as control; C1(K)N, C1 grown in the presence of KBAB4 flushed with N_2_. (**D)** 42C, monoculture of LMG 2342^T^ as control; 42N, monoculture of LMG 2342^T^ flushed with N_2_; 42(K)C, LMG 2342^T^ grown in the presence of KBAB4 as control; 42(K)N, LMG 2342^T^ grown in the presence of KBAB4 flushed with N_2_.

The enumeration of viable cells from KBAB4 grown in monoculture revealed that, irrespective of the conditions, both the control (KC) and the treated (KN) cultures followed a similar growth trend during the first 24 h, marking the end of the exponential growth phase. In the subsequent stationary phase, the control culminated at approximately 8.5 log-units during the remaining 5–6 days (Figures 1A,B). In contrast, under the N_2_ treatment, KBAB4 (KN) gradually decreased after 24 h; the level of the surviving cells was reduced by four log-units (approximately 10,000-fold) compared to the control (KC), after 5 or 6 days of flushing at 25°C (Figures 1A,B).

### EM analyses on the ultrastructure of *B. Weihenstephanensis* cells at 25°C reveal the bactericidal effect of N_2_ gas flushing

#### SEM

**Figure 2:**
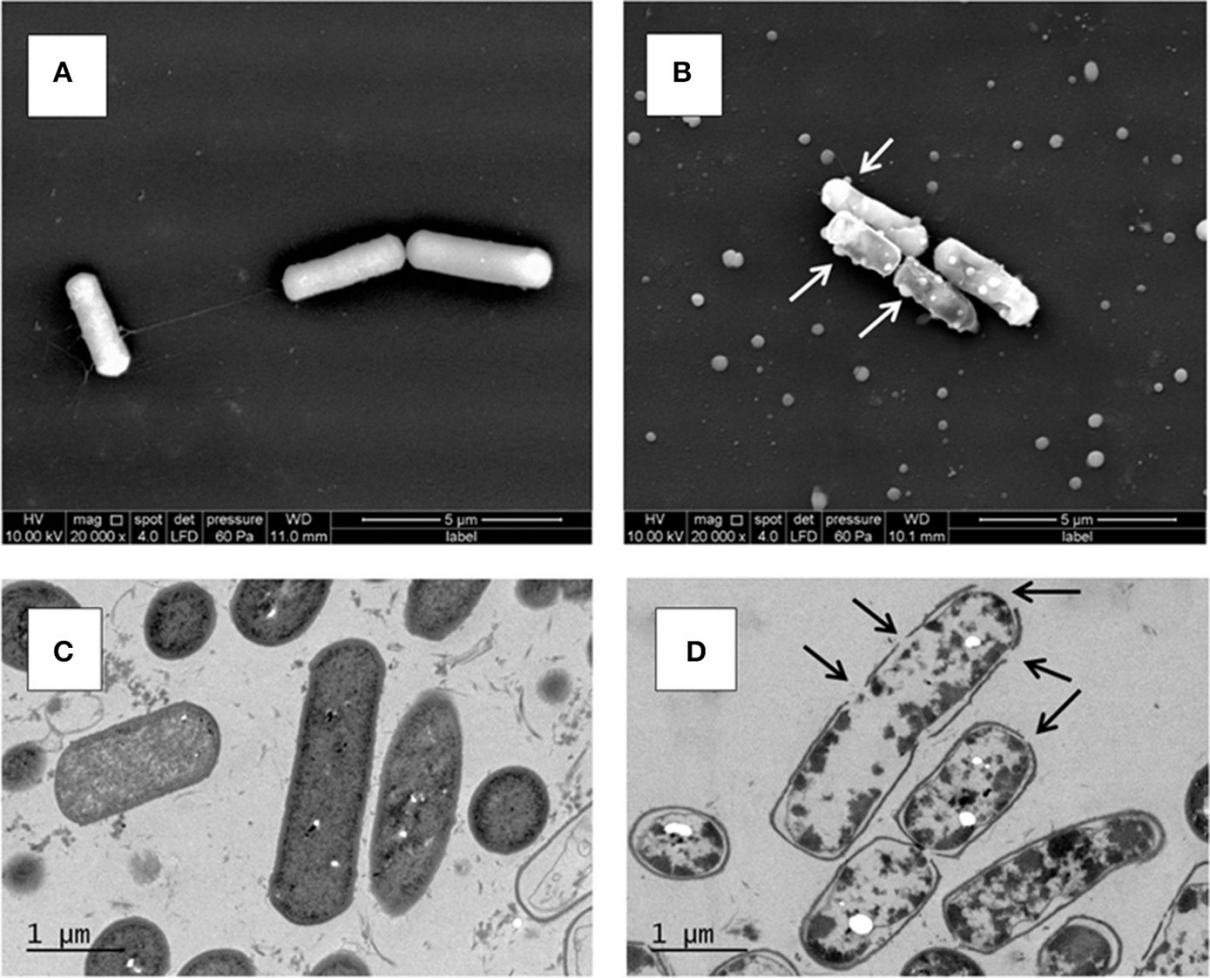
**Ultrastructure of *B. weihenstephanensis* KBAB4 cells grown at 25°C in BHI broth for 6 days**. **(A,B)** SEM micrographs of cells from **(A)** the control culture (KC in Figure 1A) or **(B)** the N_2_ flushed culture (KN in Figure 1A). White arrows indicate some of the cell wall-associated vesicle or bubble structures. **(C,D)** TEM micrographs of thin-sectioned cells from **(C)** the control culture (KC in Figure 1A) or **(D)** the N_2_ flushed culture (KN in Figure 1A). Black arrows indicate hole-like disruptions in the cell wall.

At 25°C, after 6 days of growth (Figure 1A), the untreated KBAB4 cells (KC) from the control culture were rod-shaped, approximately 2–4 μm long, turgid, and displayed an intact smooth surface (Figure 2A). In contrast, at the end of the N_2_ flushing treatment (Figure 1A), cells that were flushed (KN) were slightly shorter and thicker, with a more irregular and shrunken cell morphology. Most strikingly, many bubbles or vesicle-like round structures of varying sizes protruded from the cell surface; in addition, free, small, round particles of varying sizes were found outside the cells (Figure 2B).

#### TEM

The unflushed control cells (KC) obtained from the end point of the experiment at 25°C after 6 days of growth (Figure 1A) revealed a regular ultrastructure in both longitudinal and transversal sections. The cell wall and cytoplasmic membrane were intact and integral for most cells, even though some dead cells were visible in the preparation (Figure 2C). In contrast, the cells of the N_2_ flushed culture (KN) grown at 25°C for 6 days (Figure 1A), had lost their regular shape and were deformed. Moreover, the staining intensity of the cytoplasmic space was irregular and reduced compared to the control cells (Figure 2D). The majority of the N_2_ flushed cells were seriously damaged and shrunken; the cytoplasmic membrane was separated from the cell wall. The remnants of the cytoplasm were aggregated. Most significantly, the cell wall and possibly the cell membrane were locally disrupted in many places in a single cell. The presence of holes in the cell wall correlated with the appearance of the cells, which were more or less empty of their cytoplasmic content. Only a very limited number of cells retained their integrity (Figure 2D).

### Inhibition of the growth of pseudomonas strains under N_2_ flushing in monoculture at 25°C

Contrary to KBAB4 (K), where KN grew as fast as KC, the monocultures of the pseudomonads C1 (C1) and LMG 2342^T^ (42) revealed that the N_2_ treatments (C1N and 42N) inhibited the growth of both strains; the exponential growth phases were delayed compared to the corresponding controls (C1C and 42C) (Figures 1C,D). For example, 10^8^ cfu/ml was reached at approximately 2.5 d by C1N, less than one day by C1C, almost 1 d by 42C and nearly 5 d by 42N (Figures 1C,D).

### N_2_ flushing modifies bacterial interactions in co-culture at 25°C

The N_2_ flushing treatment did not impact the growth trend of KBAB4 co-cultured with C1 (K(C1)N); KN and K(C1)N had similar bacterial counts on the same sampling days (Figure 1A). In contrast, for the control co-culture (K(C1)C), the number of KBAB4 cells gradually decreased after 2.5 d and was up to three log-units lower than the level of KBAB4 in the control of the monoculture (KC) after 6 d (Figure 1A). The growth of KBAB4 co-cultured with LMG 2342^T^ remained mostly unaffected by the presence of LMG 2342^T^ in either control (K(42)C) or N_2_ -flushed (K(42)N) co-culture conditions (Figure 1B).

The strain C1 was more sensitive to the N_2_ flushing treatment when grown simultaneously with KBAB4 (Figure 1C). As a single culture, after 2.5 d growth, C1 increased up to eight log units under the N_2_ treatment (C1N), whereas in co-culture with KBAB4, the growth of C1 (C1(K)N) was repressed, as the bacterial levels remained stable at approximately six log-units for five consecutive days (Figure 1C). The presence of KBAB4 did not influence the growth of LMG 2342^T^ in either control (42(K) C) or N_2_ -flushed (42(K) N) cultures (Figure 1D).

### Effect of N_2_ flushing on the growth of bacterial mono- and co-cultures at 15°C

**Figure 3:**
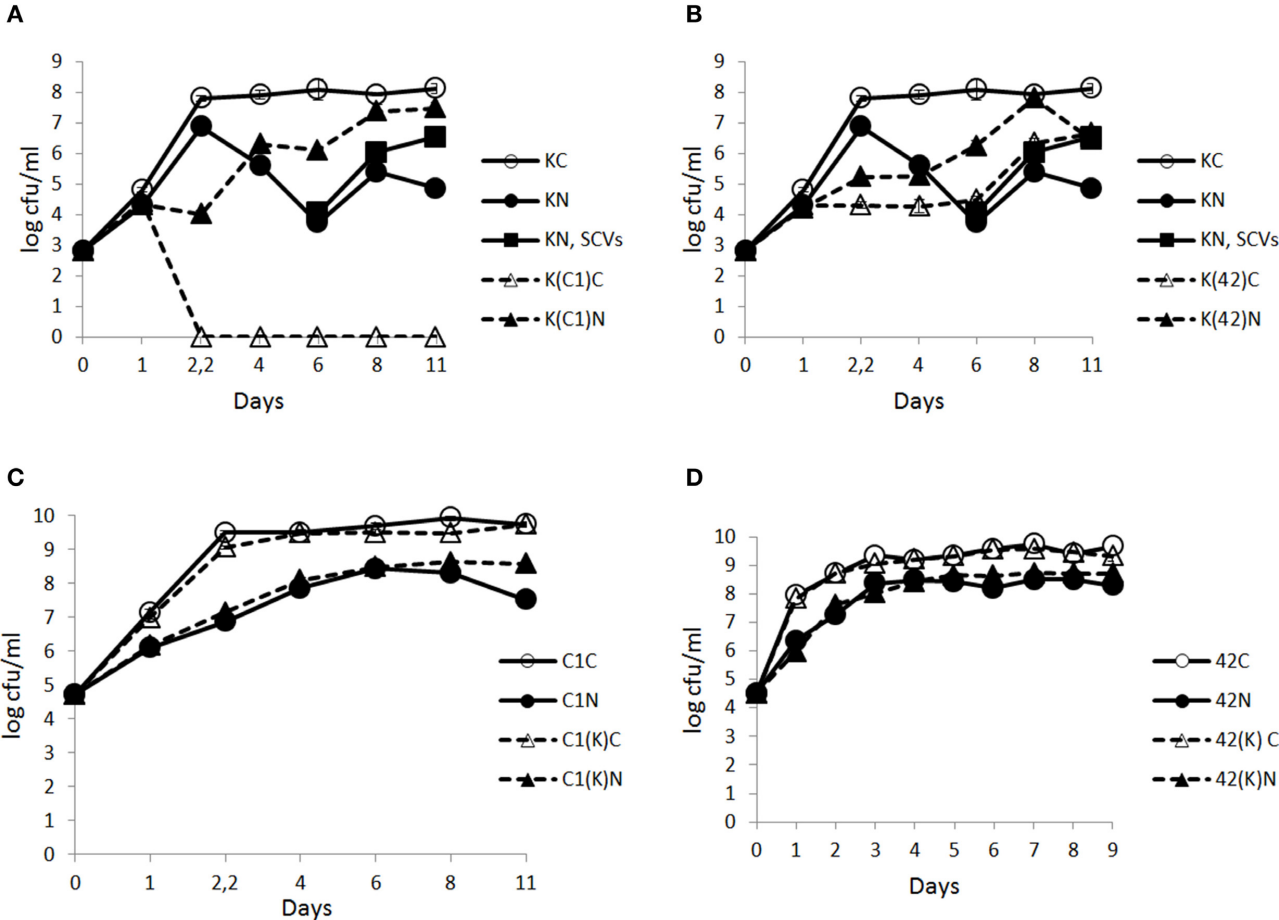
**Time-course analysis of the number (cfu/ml) of *B. weihenstephanensis* KBAB4 cells (K) established as mono- or co-cultures in BHI broth at 15°C with (A) a *Pseudomonas* raw milk isolate C1 (C1) or (B) *P. tolaasii* LMG 2342^T^ (42) together with the emergence of small colony variants (SCVs) in the KBAB4 strain monoculture**. Similarly, the number (cfu/ml) of **(C)** *Pseudomonas* strain C1 (C1) or **(D)** LMG 2342^T^ (42) cells as mono- or co-cultures with *Bacillus* strain KBAB4 under continuous pure N_2_ gas flushing (N) together with the control conditions **(C)**. Error bars indicate standard deviations. **(A,B)** KC, monoculture of *B. weihenstephanensis* KBAB4 as control; KN, monoculture of *B. weihenstephanensis* KBAB4 flushed with N_2_; KN, SCVs: small colony variants of KBAB4; K(C1)C, KBAB4 grown in the presence of C1 as control; K(C1)N, KBAB4 grown in the presence of C1 flushed with N_2_; K(42)C, KBAB4 grown in the presence of LMG 2342^T^ as control; K(42)N, KBAB4 grown in the presence of LMG 2342^T^ flushed with N_2_. **(C)** C1C, monoculture of the raw milk isolate C1 as control; C1N, monoculture of the raw milk isolate C1 flushed with N_2_; C1(K)C, C1 grown in the presence of KBAB4 as control; C1(K)N, C1 grown in the presence of KBAB4 flushed with N_2_. **(D)** 42C, monoculture of LMG 2342^T^ as control; 42N, monoculture of LMG 2342^T^ flushed with N_2_; 42(K)C, LMG 2342^T^ grown in the presence of KBAB4 as control; 42(K)N, LMG 2342^T^ grown in the presence of KBAB4 flushed with N_2_.

In the control conditions, KBAB4 in monoculture (KC) achieved its exponential growth phase at approximately 2.2 d (Figure 3A); it then entered the stationary phase and the cell counts remained constant until the end of the experiment (day 11). Under the N_2_ gas flushing treatment, KBAB4 in monoculture (KN) showed a peculiar cyclic growth trend, where total cell counts decreased or increased over time, without reaching the levels observed for the control on the corresponding sampling days (Figure 3A). At approximately 6 days of N_2_ flushing, the viable cell counts were the lowest and the culture was translucent. But, at the same sampling point, concomitant with the normal large colony type (a size similar to the colonies on the control plates of approximately 3–4 mm in diameter on BHI agar after 24 h incubation at 30°C), small colony variants (SCVs) appeared (a colony size less than 1.5 mm to pin-point) and increased over the course of the experiments (Figure 3A, Table 1).

**Table 1:** **Small colony variants as log cfu/ml that were enumerated from monocultures of *B. weihenstephanensis* KBAB4 (K) or from co-cultures with the *Pseudomonas* raw milk isolate C1 (C1) or with *P. tolaasii* LMG 2342^T^ (42) at 15°C in BHI broth**.

**EXPERIMENT A:**	**INITIAL LEVELS: C1 = 5.5 10^4^ cfu/ml; K = 6500 cfu/ml; 42 = 1.5 10^4^ cfu/ml**										
	**INITIAL RATIOS: C1/K = 8.5; 42/K = 2.3**										
**Day/Sample**	**≤5 d**		**6 d**	**7 d**		**8 d**	**9 d**		**10 d**		**12 d**
KC	0		0	0		0	0		0		0
KN	0		5.01 ± 0.07	6.17 ± 0.06		6.03 ± 0.06	7.08 ± 0.19		7.42 ± 0.10		7.23 ± 0.07
K(C1)C	0		6.35 ± 0.18	6.12 ± 0.07		6.08 ± 0.09	5.96 ± 0.03		0		0
K(C1)N	0		5.92 ± 0.10	6.85 ± 0.10		7.26 ± 0.06	0		0		5.99 ± 0.08
K(42)C	0		5.02 ± 0.07	5.84 ± 0.13		6.83 ± 0.04	0		0		0
K(42)N	0		6.52 ± 0.09	6.68 ± 0.14		7.05 ± 0.12	7.30 ± 0.18		0		6.79 ± 0.18
**EXPERIMENT B:**	**INITIAL LEVELS: C1 = 1.1 10^5^ cfu/ml; K = 5750 cfu/ml; 42 = 3.3 10^4^ cfu/ml**										
	**INITIAL RATIOS: C1/K = 18.8; 42/K = 5.7**										
**Day/Sample**	**≤4 d**		**5 d**	**6 d**		**7 d**		**8 d**	**9 d**		
KC	0		0	0		0		0	0		
KN	0		5.38 ± 0.02	6.43 ± 0.04		7.18 ± 0.03		7.27 ± 0.06	7.39 ± 0.04		
K(C1)C	0		0	0		0		0	0		
K(C1)N	0		5.30 ± 0.13	6.86 ± 0.04		7.22 ± 0.11		7.06 ± 0.38	6.95 ± 0.02		
K(42)C	0		5.02 ± 0.05	5.91 ± 0.06		6.07 ± 0.03		6.84 ± 0.02	6.82 ± 0.03		
K(42)N	0		6.00 ± 0.10	7.07 ± 0.18		7.11 ± 0.10		6.89 ± 0.15	6.62 ± 0.12		
**EXPERIMENT C:**	**INITIAL LEVELS: C1 = 5.4 10^4^ cfu/ml; K = 685 cfu/ml; 42 = 7.4 10^4^ cfu/ml**										
	**INITIAL RATIOS: C1/K = 78; 42/K = 108**										
**Day/Sample**		**≤4 d**			**6 d**		**8 d**			**11 d**	
KC		0			0		0			0	
KN		0			4.03 ± 0.47		6.05 ± 0.19			6.54 ± 0.03	
K(C1)C		0			0		0			0	
K(C1)N		0			0		0			0	
K(42)C		0			4.97 ± 0.02		0			0	
K(42)N		0			6.81 ± 0.10		0			0	

*These values were determined from three independent experiments (A, B and C) with variable initial inoculation levels and ratios of the strains. C and N correspond to the control and N_2_-flushed cultures, respectively*.

The appearance of SCVs was consistent with the N_2_ flushing treatment observed in all experiments at 15°C when KBAB4 was grown as a single culture, irrespective of the initial inoculum level; SCVs were initially lowest in the culture where KBAB4 was initially present at the lowest inoculum level (Figure 3A, Table 1). SCVs were not present in the control (KC) and appeared after 5–6 d of N_2_ flushing. The SCVs constantly grew over time by 2–2.5 log-units until the end of the experiment. After 6 d, SCVs outnumbered the original large colony morphotype and approached control levels (KC) after 8 d. Accordingly, at the end of the flushing treatments, the SCV phenotype predomined (Figure 3A).

### SEM analysis of *B. weihenstephanensis* KBAB4 cell morphology revealed changes in cell organization at the end of N_2_ gas flushing at 15°C

**Figure 4:**
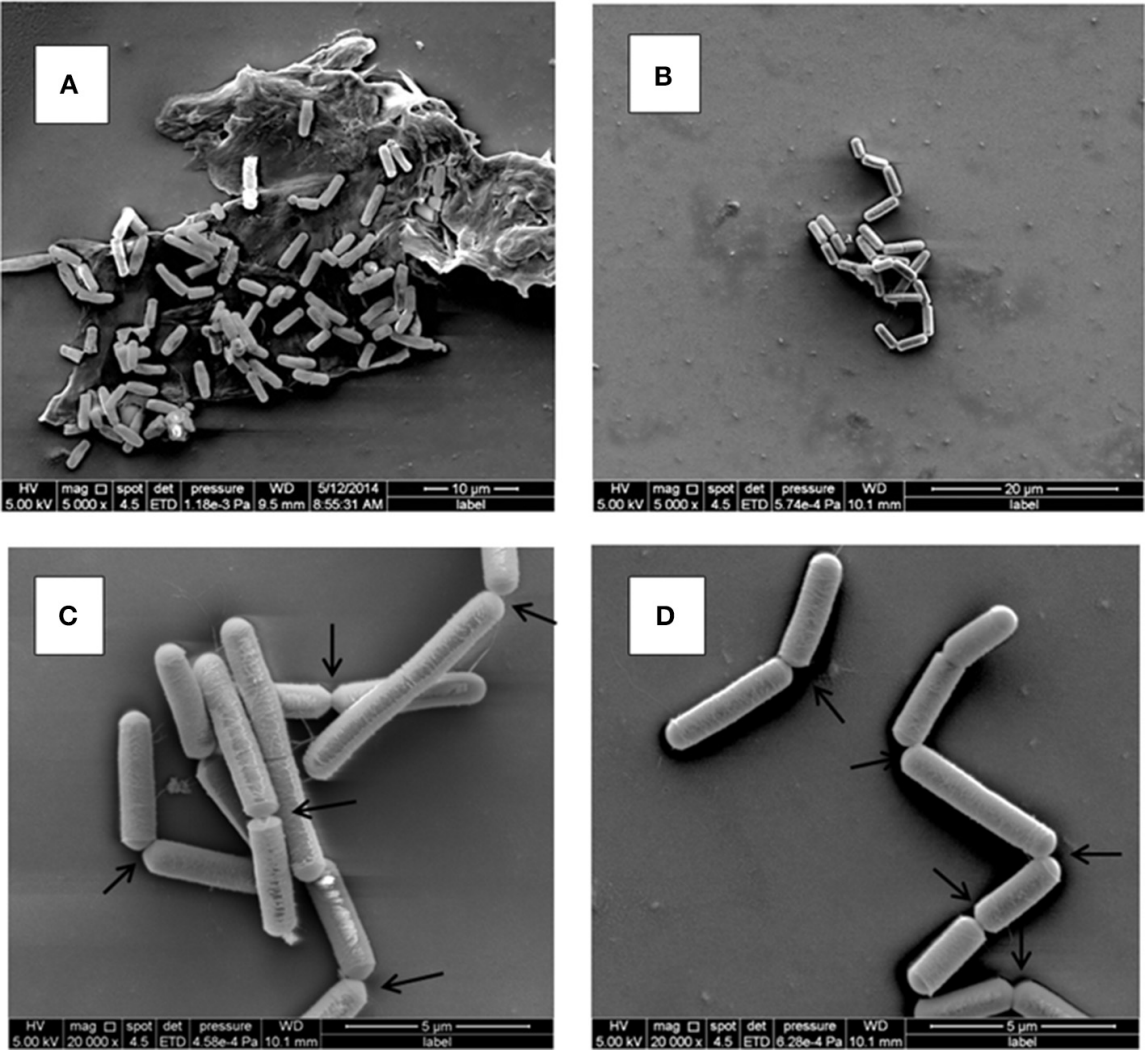
**Ultrastructure of *B. weihenstephanensis* KBAB4 cells grown at 15°C in BHI broth for 11 days**. SEM micrographs of cells from **(A)** the control culture (KC in Figure 3A) or **(B–D)** the N_2_-flushed culture (KN in Figure 3A). Arrows show some of the connections between the cells, organized as chains, indicating impaired cell separation.

KBAB4 cells of the control monoculture (KC) grown for 11 d at 15°C (Figure 3A) were of regular size and shape and were mostly organized as separate cells, but sometimes also as pairs where two cells were attached end-to-end (Figure 4A). In contrast, KBAB4 cells of the N_2_-flushed monoculture (KN) grown for 11 d at 15°C (Figure 3A) were smaller, both in length and in width, but most significantly, these cells were primarily grouped in chains with very few free single cells (Figure 4B). As shown at a higher magnification, KBAB4 cells were attached to each other at their polar ends (Figures 4C,D).

### N_2_ gas flushing inhibits also the growth of *Pseudomonas* at 15°C

At 15°C, the growth of *Pseudomonas* strain C1 in monoculture was inhibited by the N_2_ flushing treatment. C1C and C1N reached 10^8^ cfu/ml after about 1.5 d and 4 d, respectively (Figure 3C). Similarly, inhibitory effects were recorded for *P. tolaasii* LMG 2342^T^ as a single culture under the N_2_ treatment; 42C and 42N reached 10^8^ cfu/ml at 1 d and 2.2 d, respectively (Figure 3D).

### N_2_ gas flushing modifies bacterial interactions in co-culture at 15°C

At 15°C, in the controls, KBAB4 (K(C1)C) was no longer detected at different time points, when it was co-cultured with C1. After 2.2 d, it was completely eliminated in one co-culture with C1 (Figure 3A); the corresponding disappearance time points were 4 d and 12 d in the two other experiments (data not shown). Under the N_2_ flushing treatment, the growth of KBAB4 (K(C1)N) first followed KN but then entered a cyclic trend after 1 d, which was asynchronous compared to KN (Figure 3A). After 4 days, the growth of K(C1)N proceeded by stages and supplanted KN.

In the control co-culture of KBAB4 and LMG 2342^T^, the growth of KBAB4 was strongly inhibited after 1 day, while K(42)C did not achieve the levels of the control monoculture KC (Figure 3B). Surprisingly, under N_2_ treatment, the inhibitory effect of LMG 2342^T^ on KBAB4 was reduced, because the levels of K(42)N were greater than K(42)C (Figure 3B).

The emergence of SCVs was not restricted to N_2_-flushed monocultures of KBAB4 (Figure 3A, Table 1), but was also observed when KBAB4 was grown in co-culture with either C1 or LMG 2342^T^ (Table 1). For several conditions, the rate of increase of SCVs in co-cultures exceeded that observed in monocultures, for which a more progressive trend was recorded. SCVs were only recorded transiently from days 6 to 9 for the K (C1)C cells while KBAB4 decayed in the co-culture after 10 days (Experiment A in Table 1). No SCVs were recorded for the two other conditions when KBAB4 was eliminated from the co-culture with C1, after 2.2 d and 4 d. SCVs were encountered for K(C1)N in both conditions where the initial levels of KBAB4 were the highest, when the ratios of initial bacterial levels C1/K were 8.5 and 18.8 (Experiments A and B in Table 1), respectively, but not in the condition where KBAB4 was the minor component (when the ratio C1/K was 78, Experiment C in Table 1).

For the co-cultures KBAB4/LMG 2342^T^, both controls and the N_2_-flushed cultures had transient SCVs (at sampling day 6, Experiment C in Table 1) or for several consecutive days (Experiments A and B in Table 1). As shown in Table 1, during the first three days of the emergence of SCVs, the levels of SCVs in N_2_-flushed cultures (K(42)N) exceeded the levels of the corresponding controls (K(42)C). However, when KBAB4 was the minor component of the co-culture (the initial ratio 42/K was 108, Experiment C in Table 1), SCVs were absent from the control and were ephemeral in the treated culture. In contrast, the two other conditions had initial 42/K ratios of 2.3 and 5.7 (Experiments A and B in Table 1) and SCVs were more persistent, in either control or N_2_-flushed cultures.

### N_2_ gas flushing does not promote sporulation of KBAB4 in mono- or in co-cultures

The enumeration of viable colonies after heat-treatment of the different cultures revealed that at 25°C, under the N_2_ gas flushing treatment, KBAB4 was unable to sporulate in either mono- or co-cultures (Table 2).

**Table 2:** **Spore levels as log cfu/ml^a^ on BHI agar for *B. weihenstephanensis* KBAB4 (K) grown in mono- or in co-cultures with the *Pseudomonas* raw milk isolate C1 or with *P. tolaasii* LMG 2342^T^ (42) at 25°C in BHI broth**.

**Experiment**	**E.1**			**E.2**			**E.3**		
**Sample/Day**	**0**	**3**	**5**	**0**	**1.5**	**5**	**0**	**2.5**	**6**
KC	0	0	3.9	0	0	3.2	0	2.3	2.7
KN	0	0	0	0	0	0	0	0	0
K(C1)C		ND^b^		0	2.7	3.3	0	2.0	3.0
K(C1)N		ND^b^		0	0	0	0	0	0
K(42)C	0	3.9	4.6	0	3.7	3.2	0 3	3.0	4.3
K(42)N	0	0	0	0	0	0	0	0	0

*C and N correspond to the control and to N_2_-flushed cultures, respectively*.

a culture samples were heat treated for 80 °C/12 min

b *ND, not determined*.

In the corresponding controls, the presence of spores was detected in monocultures of KBAB4 or when KBAB4 was associated with either C1 or LMG 2342^T^ in all three experiments (Table 2). At 15°C, no spores were detected in the controls and flushed cultures at the intermediate or final stages of the experiments, irrespective of the types of mono- or co-cultures of KBAB4.

## Discussion

All previous studies, that investigated the possibility of treating raw or pasteurized milk with pure N_2_ gas, examined the impact of the treatments on some bacterial types retrieved on certain agar types (Murray et al., 1983; Dechemi et al., 2005; Munsch-Alatossava et al., 2010a,b, 2013). Scarce but repeated observations allowed for an incomplete identification of the targeted bacteria among Gram-(+) or Gram-(−) representatives, and prompted the present study to investigate whether at the cellular level, the treatment would have deleterious effect on certain bacterial types.

*Pseudomonas* retrieved from raw milk samples, at different stages of the cold chain of milk storage, are quite diverse. Several isolates, including the raw milk isolate C1, were ascribed to *P. tolaasii* species based on phenotypic characterisation (Munsch-Alatossava and Alatossava, 2006). Partial 16S rRNA gene sequencing suggested that C1 could be a member of *Pseudomonas fluorescens* species (unpublished data). *P. tolaasii* is a common mushroom pathogen that produces a white line in agar when streaked in front of a *Pseudomonas* “*reactans*” bacterial type (Wong and Preece, 1979); this reaction is due to the interaction of the tolaasin toxin and the white line-inducing principle (WLIP), two cyclic lipodepsipeptides (CLPs) synthesized by these bacteria (Mortishire-Smith et al., 1991; Nutkins et al., 1991). When C1 was tested for tolaasin production in the white line reaction, it did not behave like *P. tolaasii* or *P. tolaasii* -like strain (Munsch et al., 2002); on the contrary it yielded a typical white line when tested against *P. tolaasii* LMG 2342^T^ (data not shown). These result suggests that C1 is a CLP producer, has a *P*. “*reactans*” behavior and produces a WLIP type of lipodepsipeptide. The bacterium *Pseudomonas* “*reactans*” was recently re-classified as *P. fluorescens* (Ghequire et al., 2013). The incidence of CLP producers in milk is not well known, but a recent report demonstrated the implication of CLP production in false positive results when screening for antibiotic residues in milk (Reybroek et al., 2014). Considering that tolaasin had an inhibitory effect on *Bacillus subtilis* and *B. megaterium* (Rainey et al., 1991) and that C1 is also a CLP producer, both representatives of two different types of CLPs producers, C1 and *P. tolaasii* LMG 2342^T^, were selected in this study.

As monocultures, the growth of the Gram-(−) representatives C1 and LMG 2342^T^ in BHI broth was inhibited under the N_2_ flushing at both temperatures (25 and 15°C) (Figures 1C,D, 3C,D). The temperature had the opposite effect on C1 and LMG 2342^T^ under the N_2_ treatments. Irrespective of the initial bacterial levels or the growth temperature, under the control conditions, both C1 and LMG 2342^T^ reached 10^8^ cfu/ml after 1 day of growth. Under N_2_ flushing, LMG 2342^T^ reached 10^8^ cfu/ml after 5 days at 25°C, but after 2.5 d at 15°C. In contrast, C1 reached 10^8^ cfu/ml under N_2_ flushing at 2.5 d at 25°C, and at 6 d at 15°C. Altogether, the temperature modulated growth inhibition without inducing cell death.

The determinant role of the temperature on cell behaviors under N_2_gas flushing was most obvious in the case of the Gram-(+) representative *B. weihenstephanensis* KBAB4 strain grown in monoculture (Figures 1A,B, 3A). The N_2_ gas flushing of the single culture (KN) was associated with a temperature-dependent effect on KBAB4, where rapid growth (as rapid as the control monoculture KC) until the end of exponential phase was followed by an immediate cell death phase at 25°C (Figure 1A). Interestingly, the decrease in the number of vegetative cells was not correlated with sporulation; under the N_2_ treatment sporulation was prohibited, irrespective of whether KBAB4 was grown in mono- or co-cultures (Table 2). The observation that N_2_ flushing does not promote sporulation was previously reported for psychrotolerant bacteria present in pasteurized milk samples (Munsch-Alatossava et al., 2013).

The examination of the relative numbers of bacterial cells showed that, at 25°C, in co-culture with either C1 or LMG 2342^T^ (irrespective the initial levels, whether KBAB4 is the major or minor component in dual cultures) under the N_2_ treatments, KBAB4 more rapidly dominated at the early growing stages, compared to controls, until 24 h at which stage both pseudomonads largely outcompeted KBAB4 (Figure 1).

Conversely, at 15°C under N_2_ flushing, KBAB4 (KN) followed the control (KC) only in the early phase of the exponential growth; growth was then slowed and finally declined and reached a lower level of viable cells (Figure 3A). Notably, during the collapse period, KBAB4 warded off and exhibited another survival strategy by directing the bacterial genetic machinery to the emergence of SCVs, implying a morphotype of smaller colonies compared to the regular large colony morphotype of KBAB4 (Figure 3A, Table 1). At 15°C, *B. weihenstephanensis* KBAB4 responded to the stress, of chemical (N_2_ flushing) or biological (bacterial antagonism) nature, by the production of SCVs. In the case of bacterial antagonism, the level of SCVs was dependent on the initial inoculum level of KBAB4, and on the relative abundance of KBAB4 in the co-culture. If KBAB4 was most disadvantaged, with ratios of C1/KBAB4 = 78 and LMG 2342^T^/KBAB4 = 108, lower numbers of SCVs were observed (Experiment C in Table 1). SCVs have been described for a wide range of bacteria (Proctor et al., 2006; Schmidt Grant and Hung, 2013). In addition to the atypical colony morphology, many features are different including a slower growth rate. Recently, SCVs were described for *B. subtilis* strains that had lost their sporulation ability (Maughan and Nicholson, 2011). These changes conferred increased fitness and the alteration of metabolic pathways, including morphological changes such as the arrangements of cells in long filaments; the authors hypothesized the occurrence of different mutational events. Our SEM analysis revealed fairly homogenous KBAB4 bacterial cells (Figure 4), compared to *Staphylococcus aureus*, where the SCVs were heterogeneous, and presented impaired separation (Kahl et al., 2003). The exposure of *B. weihenstephanensis* KBAB4 to prolonged N_2_ flushing revealed cells organized in chains, not separated after cell division (Figures 4B–D). The observations that *B. subtilis* autolysin-deficient mutants show abnormally long chains (Smith et al., 2000) and that the levels of transcription of genes involved in the biosynthesis of major autolysins were reduced in the post-exponential phase in SCVs of *B. subtilis* (Maughan and Nicholson, 2011) suggest that the N_2_ flushing affects the expression of autolysins and could lead to impaired cell separation in SCVs of KBAB4 due to mutation(s) in gene(s) associated with autolysin expression. To the best of our knowledge, SCVs have not been previously described for *B. weihenstephanensis*.

The two considered *Pseudomonas* strains did not produce SCVs at 25 or at 15°C in the treatments or controls, although SCVs have been described for *P. aeruginosa* (Häussler et al., 1999).

Most probably at 15°C, C1 produces a killing factor deleterious to KBAB4, the disappearance of which in the co-cultures occurred at variable times that was seemingly dependent on the initial ratios (relative numbers of C1 and KBAB4); the more dominant C1 was, the earlier the killing occurred (Figure 3A, and data not shown). The observation that the cell counts for KBAB4 in the control co-cultured with C1 (K(C1)C) dropped over time at 25°C (Figure 1A) suggests that this factor is produced at lower amounts at 25°C than at 15°C. Alternatively or in addition, KBAB4 grown at 15°C could be more sensitive to the action of an antibacterial factor produced by C1 than when grown at 25°C due to temperature-adapted changes in biochemical and biophysical structural components, such as the lipid or fatty acid composition of bacterial cytoplasmic membrane (Chintalapati et al., 2004). Moreover the synthesis of this putative anti-*Bacillus* factor, produced by C1, seems to be prevented by N_2_ flushing at both temperatures, indicating that the N_2_ treatments can modulate bacterial antagonism (Figures 1A, 3A). Another illustration of the impact on the N_2_ treatments on bacterial antagonism can be seen in the co-culture of strains C1 and KBAB4 at 25°C, where C1 was more susceptible to the N_2_ flushing in the presence of KBAB4 (C1(K)N) (Figure 1C). Whether CLPs, either tolaasin or WLIP-type, are involved in these antagonistic interactions require further study.

What are the modes of actions of the N_2_ gas flushing treatment on a bacterial cell? Surely the exclusion of oxygen (O_2_) in the N_2_-flushed atmosphere is one difference to be considered, but is it the only one, or are there additional direct or indirect factor(s) involved in the N_2_ flushing action? *Pseudomonas* species have been categorized as strict aerobes, although they can survive in microaerophilic environments. *P. aeruginosa* can even grow in anaerobic conditions (Filiatrault et al., 2006). Interestingly, the *Pseudomonas* strains C1 and LMG 2342^T^ tolerated the anaerobic atmosphere under the N_2_ flushing treatment, although growth rates were reduced both at 25 and 15°C, presumably due to reduced energy production in the absence of oxygen (Figures 1C,D, 3C,D). *B. weihenstephanensis*, like *B. cereus*, is a facultative anaerobe. On the other hand, the growth of *B. weihenstephanensis* strain KBAB4 was not inhibited during the exponential growth phase, but entered the dead cell phase without undergoing a stationary phase. The dead cell phase of KBAB4 was most evident and final at 25°C (Figures 1A,B). Both SEM and TEM analyses revealed severe cellular damages and numerous protruding bubbles or vesicles that may correspond to expulsed portions of cytoplasm wrapped with cytoplasmic membrane (Figure 2B), indicating the presence of holes in the cell wall and cytoplasmic membrane (Figure 2D). Instead, at 15°C, the dead phase of KBAB4 was compensated by the recovery of KBAB4 growth and the emergence and further growth of SCVs of KBAB4 (Figure 3A). At 15°C, strain KBAB4, with a lower growth rate at the exponential phase, apparently had time to adapt to the environmental stress promoted by the N_2_ flushing and accordingly expressed several SOS responses, allowing at least a fraction of the cell population to survive through physiological or genetic modes of adaption (Finkel, 2006). Most likely, at 25°C, KBAB4 cells received the stress signals by the N_2_ flushing treatment to trigger the cells to the programmed cell death (PCD) pathway (Rice and Bayles, 2008; Bayles, 2014). Perhaps the redox-state (such as an intracellular NADH/NAD^+^ ratio) or an oxygen-independent signal promoted by the N_2_ flushing treatment could be involved in the control of PCD. Interestingly, a batch culture of KBAB4 in a closed tube (head space volume of air approximately 5%) did not undergo a dead phase, but exhibited exponential and stationary growth phases similar to the KBAB4 control culture (KC) at 25°C (data not shown), suggesting that the lack of O_2_ is perhaps not the only factor behind the effects of the N_2_ flushing treatment. Based on our EM analyses, after the N_2_ flushing treatment at 25°C, KBAB4 cells showed several typical ultrastructural features (Figures 2B,D), which are also associated with the bacterial PCD pathway: membrane depolarisation (by a holin), cell shrinkage and lysis (by an autolysin), and DNA condensation and fragmentation (Rice and Bayles, 2008; Bayles, 2014). Accordingly, we suggest that the bactericidal effects by the N_2_ flushing treatment could be caused by the activation of the bacterial PCD pathway.

## Conclusion

Under the N_2_ gas flushing treatment, (1) the growth of the *Pseudomonas* and *Bacillus* strains was mainly inhibited in exponential and post-exponential phases, respectively; (2) sporulation of the *Bacillus* strain was prevented; (3) bacterial interactions in co-culture conditions interfered with the susceptibility levels; (4) the growth temperature was a key determinant of behaviors under N_2_ flushing; (5) most significantly, we found that the combination of the two factors, “growth temperature” and “pure N_2_ gas flushing,” acted in a subtle way on *B. weihenstephanensis* KBAB4, as the strain formed SCVs at 15°C, but was lead to cell death at 25°C.

These observations constitute the first evidence that N_2_ gas flushing can be deleterious to bacteria.

## Author contributions

Patricia Munsch-Alatossava and Tapani Alatossava conceived and designed the experiments; Patricia Munsch-Alatossava performed the microbiological analyses; Tapani Alatossava performed the preparative work for electron microscopy studies. Both authors analyzed the data and wrote the manuscript.

### Conflict of interest statement

The authors declare that the research was conducted in the absence of any commercial or financial relationships that could be construed as a potential conflict of interest.
